# Antisense RNA based down-regulation of RNaseE in *E.coli*

**DOI:** 10.1186/1475-2859-5-38

**Published:** 2006-12-12

**Authors:** Christian Kemmer, Peter Neubauer

**Affiliations:** 1 Bioprocess Engineering Laboratory, Department of Process and Environmental Engineering and Biocenter Oulu, P. O. Box 4300, University of Oulu, FIN-90014 Oulu, Finland

## Abstract

**Background:**

Messenger RNA decay is an important mechanism for controlling gene expression in all organisms. The rate of the mRNA degradation directly affects the steady state concentration of mRNAs and therefore influences the protein synthesis. RNaseE has a key importance for the general mRNA decay in *E.coli*. While RNaseE initiates the degradation of most mRNAs in *E.coli*, it is likely that the enzyme is also responsible for the degradation of recombinant RNAs. As RNaseE is essential for cell viability and knockout mutants cannot be cultured, we investigated the possibility for a down-regulation of the intracellular level of RNaseE by antisense RNAs. During this study, an antisense RNA based approach could be established which revealed a strong reduction of the intracellular level of RNaseE in *E.coli*.

**Results:**

Despite the autoregulation of *rne *mRNA by its gene product, significant antisense downregulation of RNaseE is possible. The expression of antisense RNAs did not effect the cell growth negatively. The amount of antisense RNA was monitored quantitatively by a fluorescence based sandwich hybridisation assay. Induction by anhydrotetracycline was followed by a 25-fold increase of the detectable antisense RNA molecules per cell. The antisense RNA level was maintained above 400 molecules per cell until the stationary phase, which caused the level of expressed antisense RNAs to decrease markedly. Western blot experiments revealed the strongest reduction in the RNaseE protein level 90 min after antisense RNA induction. The cellular level of RNaseE could be decreased to 35% of the wild type level. When the growth entered the stationary phase, the RNaseE level was maintained still at 50 to 60% of the wild type level.

**Conclusion:**

In difference to eukaryotic cells, where the RNAi technology is widely used, this technology is rather unexplored in bacteria, although different natural systems use antisense RNA-based silencing, and a few studies have earlier indicated the potential of this technology also in prokaryotes. Our results show that even complicated self-regulatory systems such as RNaseE may be controlled by antisense RNA technology, indicating that systems based on antisense RNA expression may have a potential for controlling detrimental factors with plasmid-based constructs in arbitrary strains while maintaining their beneficial characteristics. The study also proved that the RNA sandwich hybridisation technique is directly applicable to quantify small RNA molecules in crude cell extracts, which may have a broader application potential as a monitoring tool in RNA inhibition applications.

## Background

Messenger RNA (mRNA) degradation is an important mechanism for controlling gene expression in all organisms. The rate of mRNA decay directly affects the steady state concentration of mRNA, thereby influencing the rates of protein synthesis. The lifetimes of mRNAs can differ significantly within a single cell and have a direct effect on message concentrations. In *E.coli *for example, different mRNAs may differ in stability by as much as two orders of magnitude. Their half-lives may range from a fraction of a minute to as long as an hour with a typical average half-life being two to four minutes [1]. In addition, the longevity of individual transcripts may vary significantly in response to growth conditions [2-5].

In *E.coli*, mRNA decay generally involves the sequential action of endonucleases and 3'-exonucleases [1]. At least three of the endoribonucleases identified in *E.coli*, RNasesE, RNase III, and RNase G have been found to initiate RNA decay [6]. For most *E.coli *mRNAs, degradation begins with the cleavage of internal AU-rich sites by RNaseE [7-12]. The cleavage products are then further degraded by endo- and exoribonucleases [13].

EndoribonucleaseE (RNaseE) is an essential protein of *E.coli *and necessary for cell viability [7,14,15]. It is assumed to be the principal endonuclease in the *E.coli *mRNA decay [16,17], while performing the first cut. The resulting fragments are then further degraded by a combination of endonucleolytic cleavage and 3'-exonucleolytic digestion [13]. While RNaseE initiates the degradation of cellular RNAs, it is most likely also responsible for the decay of the transcripts of recombinant genes. In recombinant protein production processes target mRNA instability may be one of the bottlenecks for a successful product formation. Reduction of the intracellular RNaseE level may increase the half-lives of mRNAs [15] and therefore may result in a higher product formation. As RNaseE is an essential protein, *Δrne*-strains cannot be used as host systems for the production of proteins. New systems are needed to investigate the influences of reduced RNaseE level to product formation.

RNaseE is a multifunctional endoribonuclease playing a role in the chemical degradation of bulk cellular RNA [8,9,11,18], the processing of ribosomal RNA [19,20], the decay of special regulatory messenger and structural RNAs [16,21], the control of plasmid replication [22] and the removal of poly-A-tails from transcripts [23]. It is a large multidomain protein with N-terminal ribonucleolytic activity and an RNA-binding domain. The C-terminal half of the protein contains the binding sites for all components of the degradasome and is essential for the formation of this multi-enzyme complex [24,25]. This endonuclease, 1061 amino acids in length, possesses broad cleavage-site specificity and cuts RNA in a variety of single-stranded regions that are AU-rich [26,27].

RNaseE is a 5'-end dependent endonuclease [28] and prefers 5'-monophosphorylated substrates, which are cut much more efficiently than 5'-triphosphate mRNAs. Therefore, 5'-triphosphates at nascent mRNAs protect the transcript. The cleavage products of RNaseE have 5'-monophosphates. This explains the "all-or-none" mRNA decay in *E.coli *where a slow initial cut is followed by the fast degradation of the entire mRNA [13].

*E.coli *RNaseE is essential for cell viability and either its underproduction or overproduction can impair cell growth [7,14,15]. To maintain RNaseE near its optimal cellular concentration, *E.coli *cells have evolved an autoregulatory mechanism of controlling the synthesis of this important ribonuclease [29,30]. Feedback regulation of RNaseE is mediated in *cis *by the 361nt *rne *5'-untranslated region (UTR), which could confer this property onto heterologous transcripts to which it was fused [29,31]. The 5'-UTR contains six structural domains. Of these structures only hairpin 2 is sufficient to direct efficient feedback regulation by RNaseE [31]. The autoregulation involves changes in the longevity of the *rne*transcript, whose half-life (normal ~1 min) varies in response to the level of RNaseE activity [29]. Thus, a 21-fold increase in *rne *gene dosage results in only 2.8-fold increase in the cellular protein concentration due to accelerated degradation of the *rne *mRNA. Conversely, insufficient RNaseE activity may increase the lifetime of the *rne*transcript. Therefore, expression of the *rne *gene is unusually sensitive to the cellular level of RNaseE activity. [29]

RNA interference (RNAi) is an evolutionarily conserved mechanism for the post-transcriptional regulation of gene expression. Small non-coding RNAs are used for sequence specific interaction with mRNAs, resulting in a blockage or degradation of the targeted RNA. Antisense RNA mechanisms can be found in pro- and eukaryotic organisms, but the mechanisms for achieving post-transcriptional down-regulation are diverse.

In bacteria, RNA interference (RNAi) is a nearly unexplored area of science. In eukaryotes, RNAi seems to be a common mechanism in controlling gene expression in nearly all organisms and eukaryotic gene silencing by RNAi has become a standard procedure in research. In contrast, only a few antisense RNA based regulatory mechanisms are known in prokaryotes, which control mainly the biological function of accessory genetic elements like phages, transposons and plasmids. Bioinformatics-aided searches have identified many new small RNAs (sRNA), which are mainly encoded in the intergenic regions of the *E.coli *chromosome [32]. Their function often remains unknown.

Initial applications of the antisense mechanism in bacteria were based on natural prokaryotic antisense systems. An example is the *parB *partition locus of the plasmid R1, which also provides antibiotic-free plasmid stability when it is cloned to other plasmids [33,34]. Recent studies have shown that antisense RNA based strategies also can be used to regulate the biosynthesis of global regulatory proteins and metabolites in *Clostridium acetobutylicum *[35] and *E.coli *[36,37], and that the application of an antisense RNA strategy can increase the product formation in recombinant protein production processes in *E.coli *[38].

The aim of this study was to establish a system for induced silencing of RNaseE by antisenseRNAs. By using this approach, it was possible to decrease the cellular level of RNaseE strongly. The constructed system may be a tool to decrease the innate mRNA degradation of the cell and therefore increasing the stability of target product mRNAs in recombinant processes.

## Results

### Cloning of antisense RNA expression constructs

**Figure 1:**
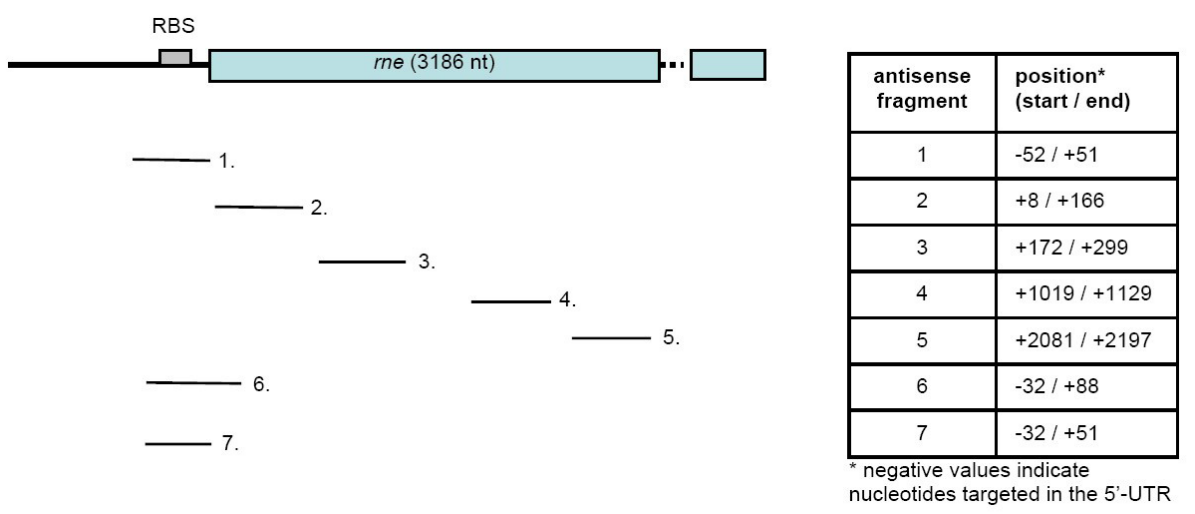
**Schematic illustration of the *rne *antisense fragment positions**. The fragment position is shown according to the *rne *mRNA. The table values indicate the position of the first and last nucleotide according to the *rne *gene sequence (*E.coli *gene accession no. EG10859). The ribosome binding site (RBS) in the 5'-UTR is marked.

A plasmid carrying two different inducible promoters were applied to determine the silencing effect of the expression of small non-coding RNaseE targeting antisense RNAs in *E.coli*. One promoter was used for the expression of small non-coding antisense RNAs, the second promoter may be later used for the gene expression of the target protein. Starting from the plasmid pP4Hcyt [39], it was necessary to introduce endonuclease restriction sites downstream of the tetracycline inducible promoter (P_tet_) before further introduction of different antisense fragments, because no restriction sites were available in the target region of pP4Hcyt. A multiple cloning site (MCS) was designed and introduced downstream of P_tet _(see Materials and Methods). Based on the DNA sequence of the *rne *gene from *E.coli *K-12 seven antisense RNA encoding fragments targeting different regions of the transcript and its 5'-UTR were amplified by PCR. The fragments were introduced into the new MCS downstream of P_tet _in antisense orientation using *Xba*I and *Bgl*II creating pANTI1 to 7, respectively. The resulting constructs were confirmed by sequencing. The sizes and location of the target regions of the non-coding antisense RNAs are shown in figure 1.

### Growth experiments

The plasmids pANTI1-7 and the control plasmid pP4Hcyt-MCS3, lacking any *rne*-mRNA targeting antisense fragment, were transformed into *E.coli *RV308. Two identical cultures of the expression strains *E.coli *RV308 carrying the antisense RNA expressing plasmids pANTI1 to 7 or the control plasmid were grown simultaneously to OD_600_= 0.1 on superbroth medium. One culture was induced by addition of aTc and the growth was monitored over 210 min.

**Figure 2:**
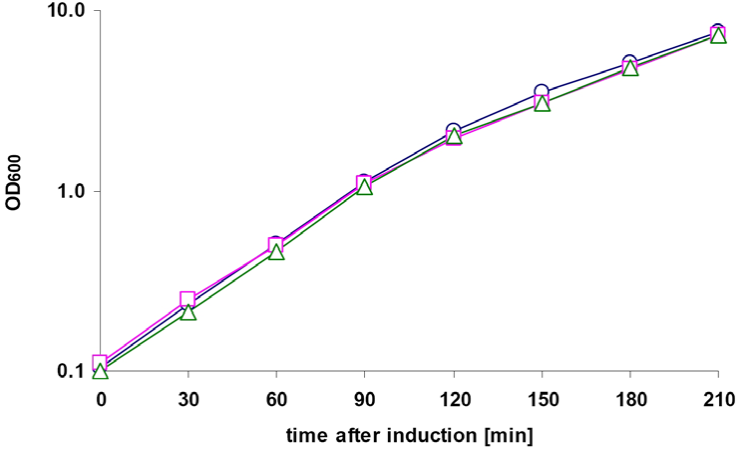
**Antisense RNA induction does not influence the growth of the host cells**. Growth curves of non-induced (circle) and induced (square) cultures of *E.coli *RV308 pP4Hcyt-MCS3 and the induced culture of *E.coli *RV308 pANTI7 (triangle).

The comparison of the growth of the control strain *E.coli *RV308 pP4Hcyt-MCS3 and all antisense RNA expressing strains in the presence or absence of inducer aTc indicated no significant difference in the cell growth. The biomass increase (OD_600_) of the cultures showed no significant differences. An example of the experimental results is shown in figure 2, where different growth curves of the non-induced and induced cultures of the control strain and the antisense RNA 7 expressing strain *E.coli *RV308 pANTI7 are presented. In order to confirm the previous results that induced antisense RNA expression has no influence on the cell growth, plating experiments were performed. Equal volumes of cell suspensions were spread on LB-plates containing ampicillin in the absence or presence of the inducer aTc, and incubated for 9 hours at 37°C. The colonies were counted and the form and size of the colonies compared. The results are shown in table 1. There was no significant difference in the number of colonies per plate. Additionally, the form and size of colonies were similar, which indicates that the expression of antisense mRNAs has no influence on the cell growth (results not shown).

**Table 1:** Colony forming units (cfu) of shake flask cultures of *E. coli *RV308 carrying various plasmids for expression of *rne *antisense RNAs with or without addition aTc

Strain *E. coli *RV 308 carrying	Plate without inducer [cfu]	Plate with aTc [cfu]
pANTI1	87	86
pANTI2	101	107
pANTI3	116	112
pANTI4	108	105
pANTI5	101	106
pANTI6	111	106
pANTI7	178	197
pP4Hcyt-MCS3	104	99

A minimum RNaseE concentration of 10 to 20% of the wild type level is sufficient to allow normal growth [15]. All expression strains carrying the control or antisense mRNA expressing plasmids showed no significant differences in the cell growth. The results of the growth experiments were promising because induced cultures showed no growth inhibition compared to the non-induced cultures. The strains, if they are expressing the antisense RNA constructs, may be therefore assumed useful for silencing RNaseE.

### Quantitative determination of antisense RNA expression

A fluorescence sandwich hybridisation assay (FSHA) as earlier described for the analysis of mRNAs [40,41] was used to determine the expression of the small non-coding antisense 6 RNA and *rne *mRNA molecules per cell. RNA molecules produced by *invitro *transcription were used as quantitative standards to determine the signal strength and background signal of different probe combinations in FSHA. The combination of two unlabelled helper probes, flanking the capture and detection probes, yielded the highest signal strength and the lowest background signal for antisense6 RNA and the *rne in vitro *standards and therefore was used for further experiments. The critical detection limit (average of blank sample added by 2.33 × standard deviation of blank sample) was below 0.1 fmol of target RNA and the signal was linear up to approximately 5 fmol in both cases.

**Figure 3:**
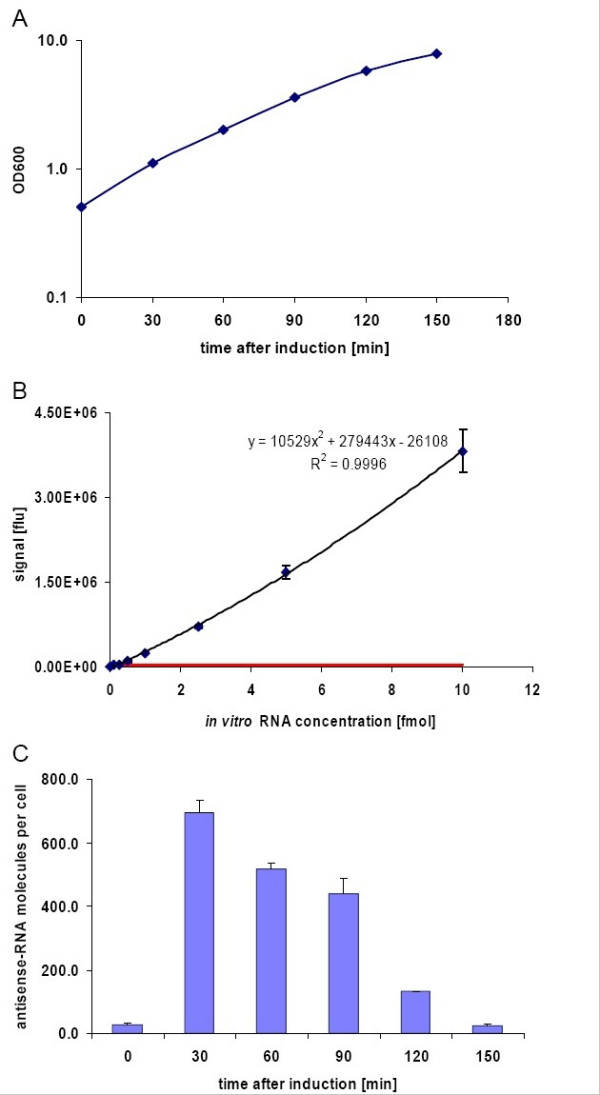
**Quantitative detection of antisense 6 mRNA by FSHA**. A)Growth curve of the culture used for sampling. B)Standard curve with antisense6 RNA *in vitro *transcripts applied for quantification of calculation of the cellular amount of antisense6 mRNA in cell extracts. The equation for the best fit of the curve is displayed and the R^2 ^value. Error bars show the standard deviation of three parallel samples. The critical level for the detection signal is indicated by a horizontal line. C)Detected antisense6 mRNA molecules per cell. Error bars indicate the standard deviation of two parallel mRNA detections in different amounts of cell extracts (2.5 and 5 × 10^6 ^cells). Induction was performed at t = 0 min with aTc.

The cellular concentration of the antisense RNA was quantified from crude cell extracts before (t = 0 min) and after induction with aTc. Therefore, as described in Materials and Methods, different concentrations of crude cell extracts at different growth points were prepared and they were analysed under identical reaction conditions on the same assay plate together with a standard curve of defined amounts of *in vitro *transcript (figures 3A and 3B). The antisense RNA amount per cell was calculated based on the standard curve and the calculated number of antisense 6 mRNA molecules per cell is shown in figure 3C.

Before induction, the level of antisense 6 mRNA detectable per cell was very low. This level corresponds to the basal expression level without addition of aTc. Already 30 min after induction with aTc the antisense 6 RNA expression showed a 25-fold increase of the target RNA corresponding to approximately 700 antisense RNA molecules per cell. At 60 to 90 min after induction, the detected antisense RNA levels were still above 400 molecules per cell. Between 90 and 120 min after induction, the culture started to pass into the stationary growth phase, characterised by reduced growth (see figure 3A). During this phase, 120 and 150 min after induction, the cellular amount of antisense RNA 6 declined markedly.

In summary, the experiments show at the example of antisense RNA 6 that the constructed vectors lead to a strong induction of *rne *antisense RNA after aTc addition.

### Detection of *rne *mRNA

To estimate the effect of antisense RNA expression on the RNaseE expression, we wanted to follow the level of *rne *mRNA in *E.coli*. Despite it had been shown earlier that the *rne *mRNA level in wild type *E.coli *cells is very low and that also the half-life of this mRNA can vary from < 40 sec to over 8 min due to the special regulatory feedback mechanism [29], we wanted to estimate whether our assay is feasible to detect *rne *mRNA under our specific experimental conditions.

Therefore, we first prepared samples from shake flask cultures according to Material and Methods. In different FSHAs, varying amounts of cell extract (1 × 10^6 ^to 5 × 10^8^) and *in vitro *standard were analysed at the same time. It was not possible to detect *rne *mRNA in the cell extracts or further purified RNA samples. Different modifications of the sample preparation procedure were tested to minimize RNA degradation in the preparation phase, such as the content of the inhibition solution, and direct sampling into pre-cooled inhibition solution and immediate freezing in liquid nitrogen, followed by slow thawing of the sample on ice. Also the amount of total RNA per well in the FSHA was increased up to 2 μg RNA per well. However, in all cases the signals obtained for *rne *mRNA were below the detection limits (data not shown).

To test whether the feedback regulatory mechanism of RNaseE is the reason for the failure of detecting *rne *mRNA in our cell extracts, the *rne *probe set was used to detect the mRNA of an inducible truncated version of RNaseE, called N-Rne, lacking the 5'-UTR of the WT transcript. This protein is expressed in the *E.coli *strain KSL2002, complementing a knockout mutation of the chromosomal *rne *gene (Lee et al., 2002).

In KSL2002 it was possible to detect the *n-rne *transcript in crude cell extracts at a basal expression (100 μM IPTG) necessary for cell viability, and after induction of the overexpression (1 mM IPTG) as well (not shown). These results demonstrate that the probe set is principally capable to detect *rne *mRNA from cell extracts. We suggest that the *in vivo rne *mRNA level is too low for regular monitoring in wild-type cells.

### Immunochemical detection of RNaseE

Finally, we wanted to demonstrate that the level of RNaseE is downregulated by the overexpressed antisense RNAs.

**Figure 4:**
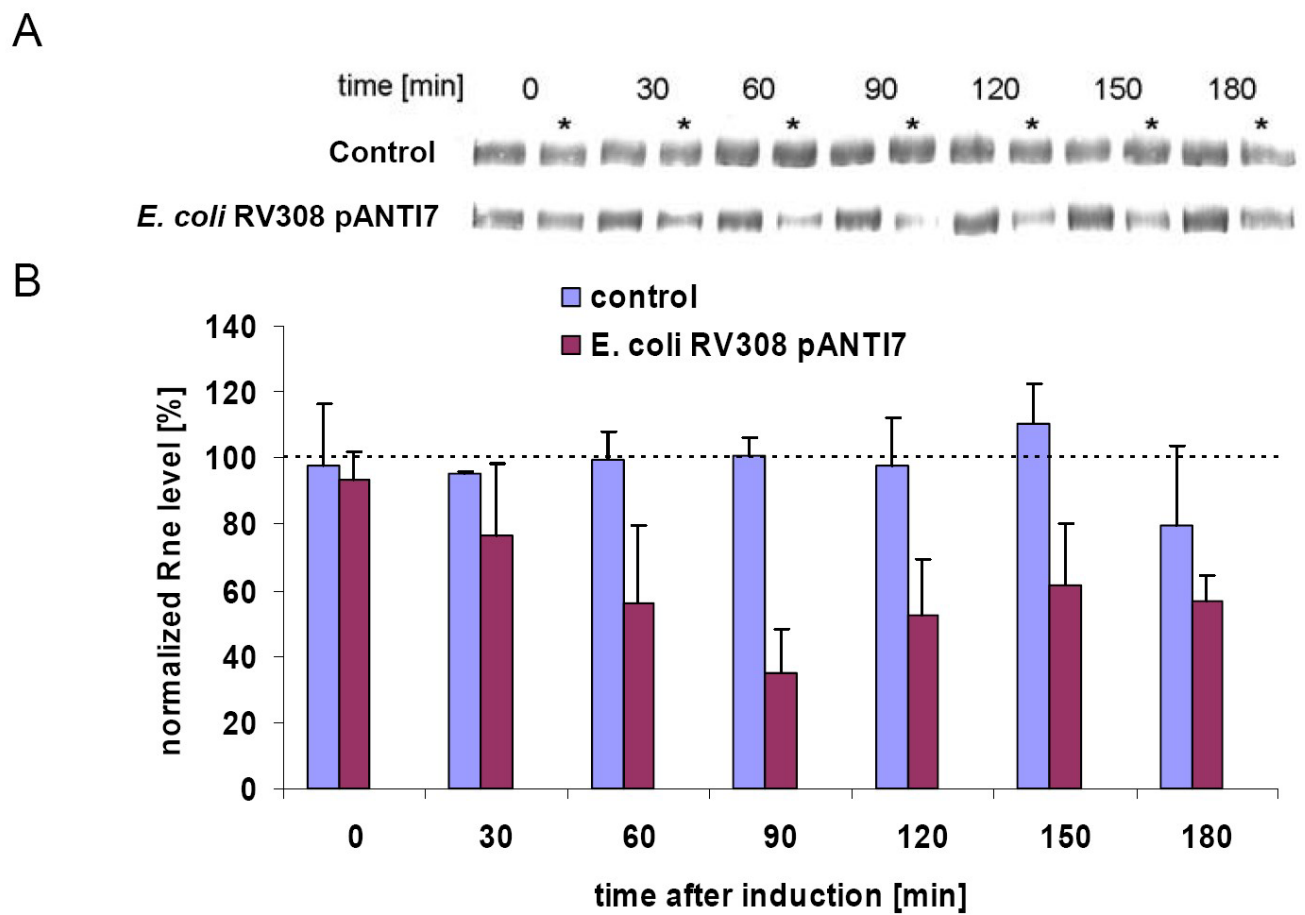
**Immunological analysis of RNaseE concentrations in *E.coli *RV308 pP4Hcyt-MCS3 (control) and pANTI7 strains**. A) Western blots of RNaseE. One of two parallel cultures of each strain was induced at time point 0 min. All lanes were loaded with equivalent amounts of total protein based on the OD_600 _of the culture. Samples of the induced cultures are marked with an asterisk. Only RNaseE bands are shown. B) Percentage of the reduction of the protein level calculated from the quantitative analysis of the RNaseE bands from the Western blots. The RNaseE level of the non-induced cultures was set to 100% (dashed line). Only results for induced cultures are shown. Error bars indicate the standard deviation of two independent and separately performed experiments.

The cultures used to monitor the bacterial growth of the non-induced and induced cultures of the *E.coli *expression strains RV308 pANTI1-7, and the control strain *E.coli *RV308 pP4Hcyt-MCS3 (see above), were used for sampling. Based on the OD_600 _of the cultures, crude cell extracts of equal cell amounts (6 × 10^7 ^cells) were prepared at different points of the growth curve. Cell extracts of both cultures of the same strains were separated on a single gel by SDS-PAGE and the content of RNaseE was determined by Western blot to compare their RNaseE level (see figure 4a).

The quantification of the Western blot results was done by using the Quantity One software (Bio-Rad). In order to achieve a meaningful comparison of different blotting data from diverse strains, the data obtained for the induced culture were normalized against the simultaneously grown non-induced culture of the same strain determined on the same blot. The RNaseE amount of the non-induced culture at each point in time was set to 100%, and the reduction in the protein level calculated. The normalized data allowed a comparison of the trace values obtained from different experiments using different strains.

The expression strain *E.coli *RV308 carrying the control plasmid pP4Hcyt-MCS3 showed no significant differences in the RNaseE content of induced and non-induced cultures, but only slight variations over the whole experimental time could be observed. The strains *E.coli *RV308 pANTI1-6 showed similar results as the control strain and no reduction in the RNaseE level in the induced culture could be determined (data not shown). Only strain *E.coli *RV308 pANTI7, expressing the antisense RNA 7 directed against the single-stranded region 3 of the 5'-UTR of the *rne *transcript, the ribosome binding site, and the beginning of the coding region (see figure 1) showed a significant reduction in the RNaseE levels. A difference was observed from 60 min after induction of the antisense RNA expression and lasted until the last sample point at 180 min after induction.

Western blot results of the RNaseE amounts in the induced and the non-induced control strains and in the antisense RNA 7 expressing strain at different time points are shown in figure 4A. Quantitative analysis of the bands revealed that the expression of antisense RNA 7 from pANTI7 reduced the RNaseE level significantly. The decrease of the RNaseE level starts slowly; the strongest reduction of RNaseE was observed 90 min after induction of the antisense RNA 7. The level of RNaseE declined to approximately 35% of the original level, measured in the non-induced culture of *E.coli *RV308 pANTI7. From 120 min after induction onwards, the level of RNaseE is maintained at 50 to 60% of the level measured in the non-induced culture. The results were reproducible in independent cultivations (see figure 4B).

## Discussion

Messenger RNAs are labile molecules whose longevity directly influences the synthesis rates of the proteins they encode. The degradation of most *E.coli *mRNAs is thought to begin with the internal cleavage in AU-rich sequences by the endonuclease RNaseE [10]. The resulting mRNA fragments are then further degraded by a combination of endonucleolytic cleavage and 3' exonucleolytic digestion [13].

During production of recombinant proteins many factors influence the formation of the target protein and target mRNA instability may be one of these bottlenecks. While RNaseE initiates the degradation of most mRNAs in *E.coli*, it is most likely also responsible for the degradation of recombinant RNAs. RNaseE is an essential enzyme necessary for cell viability [7,14,15]. Therefore, *rne *knockout strains cannot easily be used to determine the influence of RNaseE on the decay of recombinant transcripts. The aim of this study was the establishment of an antisense RNA based approach to decrease the intracellular level of RNaseE in *E.coli*. Aside from being an interesting tool for the evaluation of the effect of RNA stability, such a system also might be of practical relevance directly in recombinant processes.

In this study we could demonstrate clearly, that it is possible to decrease the amount of RNaseE significantly to about one third of its original concentration by antisense RNA targeting, despite the sensitive feed-back regulation mechanism of RNaseE on its own mRNA.

Interestingly, only one out of seven different antisense constructs, antisense 7 expressed from plasmid pANTI7, was able to reduce the intracellular RNaseE level significantly. This plasmid expressed an antisense RNA with an 83 nt long complementary sequence covering the whole single-stranded region 3 of the 5'-UTR, the ribosome binding site and the beginning of the coding region of the *rne *transcript (compare to [42]). The strongest reduction of RNaseE was observed 90 min after induction. The protein level of RNaseE decreased to approximately 35% of the wild type level. Entering the stationary phase at 120 min after induction, the RNaseE level was kept at 50 to 60% of the wild type level until the end of the experiment (see figure 4).

Interestingly, pANTI1 and pANTI6, both encoding antisense RNAs with 103 nt or 120 nt long complementary sequences respectively, covering the same region of the target RNA as pANTI7 and additionally an extended part in the noncoding region (pANTI1) or of the *rne *coding region (pANTI6), showed no effective reduction of the RNaseE level (data not shown). This might be due to the hybridisation kinetics in the crowded cytoplasm where longer molecules might be assumed to diffuse more slowly. Additionally, antisense RNA 6 includes an accessory AU-rich sequence, not present in the antisense RNA 7 which might be function as an RNaseE target site [27]. Therefore, despite being expressed at a high level (see fig. 3) the additional RNaseE cleavage site might cause faster degradation of the antisense mRNA encoded at pANTI6.

## Conclusion

Although it is still matter of detailed future investigations whether a higher stability of heterologous target RNAs can be obtained by using the antisense approach, the actual study is a further proof that down-regulation of proteins by RNA inhibition is not only feasible for regulation of arbitrary genes in eukaryotes, but also can be a powerful approach if adapted to prokaryotic cells. Especially the antisense technology may be useful tool if considered for controlling essential genes with a negative impact on target proteins, to establish industrially stable processes, which are rarely possible with knockout or conditional mutants. The advantage of using the antisense RNA technology for down-regulation of RNaseE in comparison to strains carrying the *rne131 *mutation [43] is that the feature can be easily transferred in any *E. coli *strain without influencing the fitness of the host before induction of the antisense transcript. This may be especially important for the development of industrial processes.

## Methods

### Strains

Strains used in this study are summarised in table 2. The *E.coli *strains KSL2000 and KSL2002 were kindly provided by Stanley N. Cohen (Stanford University, USA).

**Table 2:** *E. coli *strains applied in this study

***E.coli *strain**	**Properties**	**Reference**
KSL2000	*lac*Z43, *rel*A, *spo*T, *thi*-1, *rne*::*cat*, *rec*A::Tn10, carries *pBAD*-*rne*	[45]
KSL2002	*lac*Z43, *rel*A, *spo*T, *thi*-1, *rne*::*cat*, *recA*::Tn10, carries pNRNE5	[45]
RV308	Δ(*lac*)_x_*74 galPO-308*::IS *2 rpsL*	[46]
XL1 blue	*rec*A, *thi*, *sup*E44, *rel*A1, *lac*F', *proAB*, *lacI*^q^, *lacZ*ΔM15, Tn10 [Tet]	[47]

### Construction of plasmids for in vivo experiments

The plasmid pP4Hcyt [39] was used to introduce a multiple cloning site (MCS) downstream of the P_tet _while removing the gene for prolyl-4-hydroxylase (*p4h*) and its ribosome binding site. 100 pmol of two complementary synthetic oligonucleotides, MCS3fw (5'-GCTAGGATATCTTGAGCTCCAGATCTCCCGGGTTCTAGAGCGGCCGCATTTCGAATACTCGC-3') and MCS3rv (5'-GCGAGTATTCGAAATGCGGCCGCTCTAGAACCCGGGAGATCTGGAGCTCAAGATATCCTAGC-3'), were annealed in 1xSSC by heating to 95°C for 5min and a slow cool down to room temperature (restriction sites are underlined). The resulting dsDNA fragment was used for cloning.

Primers EcoRVamp_fw (5'-CAGAGCCAGCCTTCTTATTC-3') and EcoRVamp_rv (5'-GCACGGATATCGGAGTGGTAAAATAACTCTATC-3') were used to amplify a 974bp fragment of pP4Hcyt including the *Nde*I site and the tetracycline inducible promoter (P_tet_) of the plasmid. The reverse primer added an *Eco*RV restriction site 3nt downstream of the P_tet_. The *Eco*RV site was used to ligate the annealed MCS to the PCR-fragment, resulting in a 1020bp fragment, which was introduced into pP4Hcyt over *Nde*I and *Bst*BI-sites. The resulting plasmid was named pP4Hcyt-MCS3.

The new restriction sites downstream of P_tet _of pP4Hcyt-MCS3 allowed the introduction of DNA sequences used for antisense RNA expression. Based on the *rne *gene sequence of *E.coli *K12 (accession no. EG10859) different primers were designed to amplify several fragments of *rne *and its 5'-UTR with a size of 80–160bp. The primer sequences are listed in table 3. Primer pairs antisense_1 to 7fw/rv were used to amplify seven PCR fragments differing in the size and region of the *rne *gene. Isolated chromosomal DNA of the *E.coli *K-12 strain XL1-blue was used as template. The fragments were introduced in antisense direction into pP4Hcyt-MCS3, using *Xba*I and *Bgl*II sites to allow the expression of small non-coding RNAs complementary to the *rne *transcript. The different positions and sizes of the antisense fragments are illustrated in figure 1. The plasmids carrying the different antisense sequences were named pANTI1 to pANTI7.

**Table 3:** PCR primers used for amplification of antisense fragments and insertion of EcoRV restriction site into pP4Hcyt. The annealing temperature for all primers was 55°C

**Primer designation**	**Sequence 5'-3'**^1^	**Restriction site**
antisense1 fw	*ctgac*tctagaCGTTACTTTGCCCGCAGCTTAG	*Xba*I
antisense1 rv	*gtgct*agatctGGCAACGCGCAAC TCTTCCTG	*Bgl*II
antisense2 fw	*ccaag*tctagaGAATGTTAATCAACGCAACTCAGC	*Xba*I
antisense2 rv	*cgacc*agatctCAGCTTCCAGACTCGGTTCAATG	*Bgl*II
antisense3 fw	*gtagg*tctagaGTTGATTACGGCGCTGAACGTC	*Xba*I
antisense3 rv	*ggacc*agatctACTTCCTGACCTTCACGCAACAC	*Bgl*II
antisense4 fw	*cacca*tctagaGCGGCCTGATTGTTATCGACTTC	*Xba*I
antisense4 rv	*gtccg*agatctTGATTTGAATACGCGCACGGTCC	*Bgl*II
antisense5 fw	*caggt*tctagaCGAAGGCGCTGAATGTTGAAGAG	*Xba*I
antisense5 rv	*ccagt*agatctGCTCGTAACGCACTTTCTGATTG	*Bgl*II
antisense6 fw	*ggagc*tctagaAGTCGTCAATGTAAGAATAATG	*Xba*I
antisense6 rv	*ggagc*agatctCGATATCCAGGTCATACAG	*Bgl*II
antisense7 fw	*cgagc*tctagaAGTCGTCAATGTAAGAATAATG	*Xba*I
antisense7 rv	*gtgct*agatctGGCAACGCGCAACTCTTCCTG	*Bgl*II

^1 ^Sequence homologies are shown in capital letters, target sequences for restriction endonucleases are underlined, bases for protection of primer ends are italicised.

### Growth experiments

Growth experiments were performed with the *E.coli *RV308 host carrying the antisense RNA encoding plasmids pANTI1 to pANTI7 or the control plasmid pP4Hcyt-MCS3. All expression cultures were performed in three-baffled 500mL flasks containing 100mL of Luria broth (LB, Sambrook and Russel, 2001) or Superbroth (per L: 32 g trypton, 20 g yeast extract, 5 g NaCl, pH 7.5) at 37°C at 200rpm on an Infors-shaker. Bacterial growth was monitored spectrophotometrically (Ultraspec 2100 pro, Amersham) by measuring the absorbance at 600 nm (OD_600_) corresponding to a microscopical counted cell amount of about 6 × 10^8 ^cells mL^-1^. This relation is used for further calculations.

To determine the influence of the antisense RNA expression on cell growth two identical cultures were grown simultaneously in baffled shake flasks on superbroth medium to OD_600 _= 0.1. One culture was induced by addition of 0.2 μgmL^-1 ^anhydrotetracycline (aTc) and the growth was monitored over 210min. Additionally plating experiments were performed by plating equal dilutions of cell suspension on LB agar plates containing ampicillin (100 μgmL^-1^) in the presence or absence of the inducer aTc (0.2 μgmL^-1^). The plates were incubated for 9hours at 37°C and the colony amount, form and size were compared.

### Quantitative determination of antisense RNA expression

The quantitative determination of antisense RNA expression performed by a fluorescence sandwich hybridisation assay (FSHA) as described earlier [41]. Parameters for the probe design targeting antisense RNAs were previously described elsewhere [44]. The probes were placed close to each other and the capture probes were ordered 5'-biotin labelled (Metabion, Martinsried, Germany). The detection probes were 3'-digoxigenin labelled using the DIG oligonucleotide tailing kit 2^nd ^generation (Roche Diagnostics, Mannheim, Germany). In addition, unlabelled helper probes flanking the target region of the detection and capture probes were designed to reduce the secondary structure of the target antisense RNAs. Different probe sets were designed for the sequence specific detection of the *rne *transcript and antisense RNAs1 and 6 (see table 4). *In vitro *transcription was performed to produce RNAs of the DNA sequences encoding the target RNAs (see table 5). The quality of the *in vitro *transcripts was verified with the Agilent 2100 Bioanalyzer using the RNA 6000 Nano Kit (both Agilent Technologies) and the RNA concentration was determined with the Ribogreen Quantitation kit (Molecular Probes).

**Table 4:** Probes for fluorescence sandwich hybridisation

**Probe**	**Sequence 5'-3'**^1^	**label**
**Probe set for detection of *rne *mRNA**		
helper1 rne (5')	GCCGCGCTCTTCTTTATCG	-
detection probe rne	GTTAATGCCGCGCCTTTGTT	3' digoxigenin
capture probe rne	CGCCAGACTGATAAAGGTG	5' biotin
helper2 rne (3')	GGCATCAGAACCAGATAGC	-

**Probe set for detection of antisense6 mRNA**		
helper1 (5') anti6	GACCTGGATATCGAGATCTG	-
detection anti6	AGATGGGCAGCGTCTGTAT	3' digoxigenin
capture anti6	capture anti6	5' biotin
helper2 (3') anti6	AACGCAACTCAGCAGGAAG	-

^1 ^Sequence homologies are shown in capital letters. The helper probe position (3' or 5') is related relative to the target mRNA position.

**Table 5:** Primers for production for *in vitro *transcription templates

**Primer**	**Sequence 5'-3'**^1^	**Annealing temperature**	**Template**
rne amp fw	ctaatacgactcactatagggagaCAACGCAACTCAGCAGGAAG	47°C	Chromosomal DNA *E.coli*
rne amp rv	CGATCTGGTAGTGGCTGAAC	47°C	

anti6 amp fw	ctaatacgactcactatagggagaAGCTCCAGATCTCGATATCC	50°C	pANTI6
anti6 amp rv	CGCTCTAGAAGTCGTCAATG	50°C	

^1 ^T7 – promoter sequences are underlined, sequence homologies are shown in capital letters.

The resulting RNAs were used to test the signal strength and background signal of the designed probe sets and to produce a molecular weight standard as basis for the RNA quantification. The probe combinations with the highest signal and the lowest background signal were used for RNA quantifications in cell extracts. The specificity of the signal was validated by spiking cell sample, which did not contain the target RNA with different amounts of *in vitro *standards.

At different time points, the OD_600 _of a shake flask expression culture was measured and samples were taken for RNA analysis. Therefore, 1mL of the culture was collected and added directly into 125 μL of pre-cooled (-20°C) inhibition solution (95% ethanol (v/v), 5% phenol (v/v)) to inhibit the cell metabolism. The cells were pelleted by centrifugation at 10,000 × g and 4°C for 10min, and resuspended in 100 μL RNA *later*^® ^(Ambion) to protect the mRNA from degradation. Samples were stored at -70°C or kept on ice if the assay was performed at the same day.

Before preparation of crude cell extracts, the cells were collected by centrifugation at 15,000 × g and 4°C for 5 min. The pellets were resuspended in 1mL of 1 × TENS buffer and applied to tubes including 1.2 g of sterile glass beads (0.1mm diameter). The cells were disrupted with a cell homogenizer (bead mill, Thermosavant) at 6.5msec^-1 ^for 45sec and afterwards immediately cooled down on ice. The insoluble cell debris was removed by centrifugation at 15,000 × g at 4°C for 10min. The supernatant including the RNA was transferred to a new tube and processed further. Before applying to the FSHA all supernatants of the different samples were diluted to the same concentration based on the OD_600 _measurement at sampling time. For the FSHA several dilutions of the crude cell extracts were prepared in 1 × TENS buffer to obtain cell extracts from 1 × 10^10^to1 × 10^8 ^cells mL^-1^. 10 μL of the extracts were directly added to the FSHA. The data are presented either in fmol per OD_600_, or the concentration of RNA molecules as detected from the standard calibration curve is further used for calculation of the number of molecules per cell by using the Avogadro constant.

### Immunochemical detection of RNaseE

RNaseE was detected using rabbit polyclonal antiserum raised against RNaseE kindly provided by A. J. Carpousis [43]. Crude cell extracts, containing 6x10^7 ^cells (based on the OD_600 _of a culture) were prepared from samples taken from different time points during the cultivations. Therefore, cell pellets were resuspended in 30 μL of protein sample buffer and boiling for 3min. Proteins were separated by SDS-PAGE (8%) and blotted as described elsewhere [43], except that goat-anti-rabbit-AP IgG (H+L) (Caltag Laboratories) secondary antibody was used. The signal was developed by incubation of the membrane in 20mL AP-buffer (100mM Tris-Cl (pH 9.5), 100mM NaCl, 5mM MgCl_2_, 0.66% NBT, 0.33% BCIP). The western blot membranes were dried over night in the dark on Whatman paper and scanned at highest resolution (1200dpi). The RNaseE bands were quantified using Quantity One V.4.2.1 software (Bio-Rad).

## Authors' contributions

CK performed the complete experimental work of this study. The initiative for development of the antisense system for RNaseE came from PN, who also actively took part to the planning of experiments.
